# REVEL Is Better at Predicting Pathogenicity of Loss-of-Function than Gain-of-Function Variants

**DOI:** 10.1155/2023/8857940

**Published:** 2023-12-04

**Authors:** Jasmin J. Hopkins, Matthew N. Wakeling, Matthew B. Johnson, Sarah E. Flanagan, Thomas W. Laver

**Affiliations:** Department of Clinical and Biomedical Sciences, Faculty of Health and Life Sciences, University of Exeter, Exeter, UK

## Abstract

In silico predictive tools can help determine the pathogenicity of variants. The 2015 American College of Medical Genetics and Genomics (ACMG) guidelines recommended that scores from these tools can be used as supporting evidence of pathogenicity. A subsequent publication by the ClinGen Sequence Variant Interpretation Working Group suggested that high scores from some tools were sufficiently predictive to be used as moderate or strong evidence of pathogenicity. REVEL is a widely used metapredictor that uses the scores of 13 individual in silico tools to calculate the pathogenicity of missense variants. Its ability to predict missense pathogenicity has been assessed extensively; however, no study has previously tested whether its performance is affected by whether the missense variant acts via a loss-of-function (LoF) or gain-of-function (GoF) mechanism. We used a highly curated dataset of 66 confirmed LoF and 65 confirmed GoF variants to evaluate whether this affected the performance of REVEL. 98% of LoF and 100% of GoF variants met the author-recommended REVEL threshold of 0.5 for pathogenicity, while 89% of LoF and 88% of GoF variants exceeded the 0.75 threshold. However, while 55% of LoF variants met the threshold recommended for a REVEL score to count as strong evidence of pathogenicity from the ACMG guidelines (0.932), only 35% of GoF variants met this threshold (*P* = 0.0352). GoF variants are therefore less likely to receive the highest REVEL scores which would enable the REVEL score to be used as strong evidence of pathogenicity. This has implications for classification with the ACMG guidelines as GoF variants are less likely to meet the criteria for pathogenicity.

## 1. Introduction

In silico predictive tools can be used to help predict the pathogenicity of genetic variants in Mendelian disease. They are particularly useful for missense variants since these have a variable effect on the protein: even in genes where missense variants are a known cause of disease, not all missense variants will be pathogenic.

As part of the standardisation of the classification of variants causing Mendelian disease, the 2015 American College of Medical Genetics and Genomics (ACMG) guidelines stated that in silico predictive tools can be used as supporting evidence in variant classification [1] (PP3 criteria to support a variant being pathogenic and BP4 to support a variant being benign). The guidelines stratified the different lines of evidence that can be used to support a classification of pathogenic into different weights: supporting, moderate, strong, and very strong. These different lines of weighted evidence are then combined to produce an overall variant classification of either benign, likely benign, uncertain significance, likely pathogenic, or pathogenic. By classifying predictions from in silico tools as only supporting evidence, they suggested limited weight could be put on their results. However, Pejaver et al. [2], as part of the ClinGen [3] Sequence Variant Interpretation Working Group, recommended that some tools were sufficiently predictive of pathogenicity and that high scores could be used as moderate (PP3_moderate) or even strong (PP3_strong) evidence for pathogenicity.

REVEL (Rare Exome Variant Ensemble Learner) is a metapredictor—an in silico tool that combines multiple different tools and types of evidence for pathogenicity into a combined score [4]. It uses scores from 13 individual tools: MutPred [5], fathmm v2.3 [6], VEST 3.0 [7], PolyPhen-2 [8], SIFT [9], PROVEAN [10], MutationAssessor [11], MutationTaster [12], LRT [13], GERP++ [14], SiPhy [15], phyloP [16], and phastCons [17], to predict the likelihood that missense variants are pathogenic. This means that REVEL uses multiple strands of evidence to predict whether a variant is pathogenic: conservation, the difference in the physicochemical characteristics of the new amino acid compared to the reference, and the effect of the amino acid change on the structural and functional properties of the protein.

REVEL is widely used in a range of applications and can have clinical implications. Toratani et al. [18] used REVEL to highlight a potential pathogenic variant in *RUNX1* predisposing to acute myeloid leukemia in a family, which led to choosing a stem cell donor from outside the family. Schuurmans et al. [19] explored genotype-phenotype correlation in glutaric aciduria type 1 and showed that a higher REVEL score correlated with lower residual enzyme activity. Kingdom et al. [20] used REVEL to identify likely deleterious variants in genes associated with developmental disorders in order to screen the UK Biobank population cohort of 500,000 people for related phenotypes. As these examples highlight, REVEL is particularly useful as an automated assessment of pathogenicity, which can be used to take a cautious approach to pathogenicity (as in the transplant example). REVEL scores can also be easily correlated with other data, such as functional domains and enzymatic activity in the glutaric aciduria type 1 example. Finally, the scores offer the ability to classify a large number of variants in order to study the broad picture of a disease or phenotype in a large cohort where manual curation of variants may not be practical.

Gunning et al. [21] demonstrated that metapredictors, such as REVEL, provide superior predictive value over individual in silico tools. They analysed a dataset of variants from ClinVar, Human Gene Mutation Database (HGMD), and the Genome Aggregation Database (GnomAD) as well as a clinically representative dataset derived from a research/diagnostic exome and panel sequencing. REVEL had the best performance of the metapredictors tested on the results of the clinically representative dataset with an area under the receiver operating characteristic curve of 0.82. However, this study did not test whether the mechanism of action, loss of function (LoF) or gain of function (GoF), had an impact on REVEL's performance.

REVEL produces a score for a missense variant of between 0 and 1 with larger scores indicating a higher chance that the variant is pathogenic. In the paper describing the tool, the authors give two potential thresholds for considering a variant to be pathogenic: a REVEL score of 0.5, which in their dataset (a subset of HGMD) gave a sensitivity of 0.75 and specificity of 0.89, and a REVEL score of 0.75, which gave a sensitivity of 0.55 and a specificity of 0.97 [4]. Alternative thresholds were suggested by Pejaver et al. [2] who evaluated the predictive power of the REVEL scores for pathogenic and benign variants in ClinVar to recommend that a score of 0.773 could be used as moderate and a score of 0.932 as strong evidence for the pathogenicity of a variant when assigning pathogenicity using the ACMG guidelines [1]. The ability of REVEL to accurately predict the pathogenicity of a variant with relatively high sensitivity and specificity has led to the tool being incorporated into gene-specific ACMG guidelines by Variant Curation Expert Panels (VCEPs). These include *RYR1* variants causing malignant hyperthermia susceptibility [22], *ITGA2B/ITGB3* variants causing Glanzmann thrombasthenia [23], and *MYOC* variants causing glaucoma [24]—the latter of which is likely caused by a GoF mechanism.

In a previous study, SIFT and PolyPhen, two widely used in silico tools, were shown to perform less well at predicting the pathogenicity of GoF compared to LoF variants [25]. This study exploited the fact that GoF and LoF variants in three genes (*ABCC8*, *KCNJ11*, and *GCK*) cause opposing disease phenotypes (monogenic diabetes and congenital hyperinsulinism), creating a unique resource for evaluating different disease mechanisms within the same genes. In this study, we utilised this highly curated dataset to evaluate the performance of REVEL for predicting the pathogenicity of LoF and GoF variants within the same genes.

## 2. Materials and Methods

To evaluate the performance of REVEL on LoF and GoF variants, we studied the curated set of 133 pathogenic variants from Flanagan et al. [25]. We excluded two variants as one was a start-loss variant and the other was a multinucleotide variant, which REVEL is not designed to evaluate. This resulted in a set of 131 different pathogenic missense variants in the *ABCC8* (*n* = 47), *KCNJ11* (*n* = 56), and *GCK* (*n* = 28) genes (Supplementary Table 1). 66 variants were LoF while 65 were GoF. The authors of the REVEL paper [4] confirmed that the variants used in this study were not included in the training dataset for REVEL.

We downloaded the REVEL 1.3 dataset and looked up the REVEL scores for the 131 variants included in this study and evaluated the different thresholds for pathogenicity. This included REVEL scores of 0.5 and 0.75 as recommended by the authors of the tool [4]. We also investigated the REVEL thresholds recommended by Pejaver et al. [2] for using REVEL scores as different levels of evidence for pathogenicity (0.773 for moderate and 0.932 for strong).

Statistical significance was tested using Fisher's exact test.

## 3. Results

### 3.1. Using Author-Recommended REVEL Thresholds Correctly Predicts Pathogenicity of LoF and GoF Variants

The authors of REVEL recommend potential thresholds for the pathogenicity of REVEL scores of 0.5 or 0.75 depending on the context in which the tool was to be used—whether specificity or sensitivity was most important [4].

**Figure 1:**
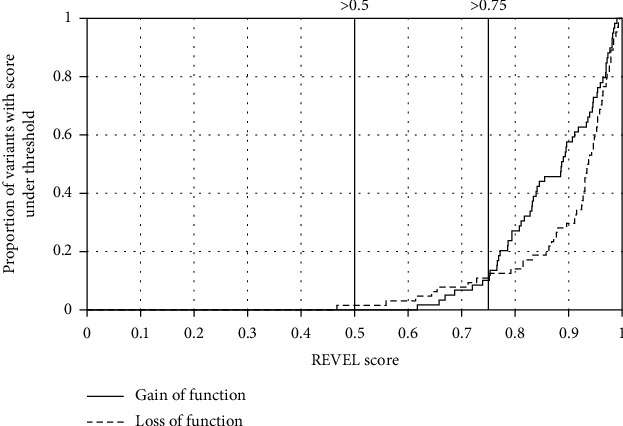
A high proportion of LoF and GoF variants meets author-recommended thresholds for pathogenicity. A graph showing the cumulative frequency of loss-of-function (LoF) and gain-of-function (GoF) variants which meet that REVEL score threshold. The REVEL score thresholds of 0.5 and 0.75 are highlighted as they were given by the tool authors as potential thresholds for pathogenicity [4].

Using a 0.5 REVEL threshold for pathogenicity, 65/66 (98%) of LoF and 65/65 (100%) of GoF variants were predicted as pathogenic. Using a 0.75 REVEL threshold for pathogenicity, 59/66 (89%) LoF and 57/65 (88%) GoF variants were predicted as pathogenic (Figure 1).

### 3.2. REVEL Scores for LoF Variants Are More Likely to Meet Criteria for Strong Evidence for Pathogenicity

**Figure 2:**
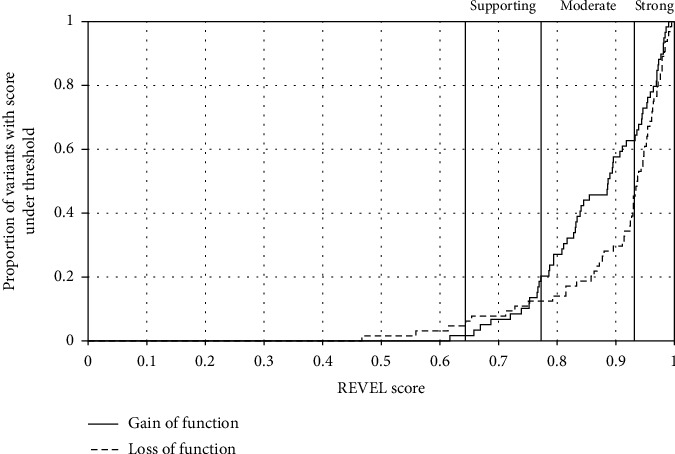
LoF variants are more likely to meet the threshold for strong evidence for pathogenicity. A graph showing the cumulative frequency of loss-of-function (LoF) and gain-of-function (GoF) variants which meet that REVEL score threshold. The REVEL score thresholds for supporting, moderate, and strong evidence are highlighted as recommended by Pejaver et al. [2].

The REVEL scores for 36/66 (55%) LoF variants meet the criteria for strong evidence (REVEL score of 0.932) as recommended by Pejaver et al. [2] (Figure 2). In contrast, only 23/65 (35%) GoF variants meet the criteria to use REVEL as strong evidence for pathogenicity (*P* = 0.0352).

Similarly, 58/66 (88%) of LoF variants meet at least the threshold for moderate evidence (REVEL score of 0.773) while 51/65 (78%) of GoF variants meet that threshold, although this difference is not statistically significant (*P* = 0.1677). 62/66 (94%) of LoF and 63/65 (97%) of GoF variants meet at least the criteria for supporting evidence (REVEL score of 0.644).

## 4. Discussion

We used a dataset of 66 LoF and 65 GoF variants to assess the performance of the widely used metapredictor REVEL for identifying pathogenic LoF and GoF variants. Using the REVEL score thresholds recommended by the authors of the tool (0.5 and 0.75) [4], REVEL performed similarly for LoF and GoF. However, when we then used the threshold recommended by Pejaver et al. [2] as strong evidence of pathogenicity (REVEL score 0.932), a greater proportion of LoF than GoF variants met the criteria for strong evidence of pathogenicity.

There is not a clear pattern for the GoF variants that met the threshold for strong: they are split between the three genes studied and spread across protein domains. For example, in *ABCC8*, using the protein domain classifications from De Franco et al. [26], the variants that met the criteria for strong were split between the highly conserved nucleotide-binding domain (*n* = 3/6), transmembrane domain (*n* = 2/6), and cytoplasmic domain (*n* = 1/6). In comparison, the variants which did not meet the criteria for strong were in the transmembrane (*n* = 3/17), cytoplasmic (*n* = 13/17), and extracellular (*n* = 1/17) domains. This suggests that while the protein domain may affect the REVEL score, it is not deterministic of whether a variant will meet the threshold to be used as strong evidence for pathogenicity.

Our results suggest that GoF variants are less likely than LoF variants to get the very highest REVEL scores that would enable them to be used as strong evidence for pathogenicity. This is in keeping with the previous findings of Flanagan et al. [25] for SIFT and PolyPhen which found that their predictive power was lower for these GoF variants. Since REVEL includes SIFT and PolyPhen-2 scores as part of its algorithm, this may explain some of this difference in performance.

In silico predictors are not a substitute for expert judgement and should not be used in isolation but as part of an overall assessment of different strands of evidence as recommended by the ACMG guidelines [1, 27]. Even if a REVEL score meets the threshold to use as strong evidence, further independent strands of evidence need to be provided for a variant to meet the ACMG criteria for pathogenicity. However, the ability to use the score from an in silico predictive tool as strong evidence of pathogenicity has important implications for variant classification. The ACMG guidelines state that two strong pieces of evidence are sufficient to declare that a variant is pathogenic [1]. A variant with a sufficiently high REVEL score would therefore only need one additional piece of strong evidence, such as in vitro functional evidence, to demonstrate pathogenicity. In contrast, if the in silico evidence can only be used as supporting evidence, then in addition to a strong piece of evidence, you would also need two moderate (such as the variant being located in a well-established functional domain without benign variation) and a second supporting piece of evidence (such as the patient's phenotype being highly specific for the disease), for example, to meet the threshold for pathogenicity. Some VCEPs have conservatively chosen to cap the use of REVEL scores for PP3 criteria to moderate [22] or supporting [23], which would mitigate the potential difference between the REVEL scores of GoF and LoF variants.

The finding that GoF variants are less likely than LoF variants to meet the score threshold to use as strong evidence of pathogenicity is important for diagnostic genetic testing of genes that cause disease via a GoF mechanism. This highlights a potential utility in developing bespoke REVEL thresholds for specific genetic conditions caused by GoF variants that would enable the scores to be used as moderate or strong evidence of pathogenicity. Indeed, Pramparo et al. [28] calculated a bespoke REVEL cutoff for pathogenic variants in *CYP27A1* causing cerebrotendinous xanthomatosis to study the prevalence and geographic distribution of the disease.

Variants in this study come from three genes where we have the expertise to confidently define whether they act via a LoF or GoF mechanism, since the two mechanisms cause the opposite phenotypes of monogenic diabetes or congenital hyperinsulinism. We did not include benign variants in the study as our aim was to evaluate the relative performance of REVEL on LoF and GoF variants rather than to assess the ability of the tool to accurately predict whether a variant was pathogenic or benign, which has already been established [4, 21]. While we expect our results to be widely applicable, we recognise that our study was limited to three disease genes, and we therefore recommend that further studies be performed on additional genes with known LoF and GoF mechanisms of pathogenicity in order to replicate our findings.

In conclusion, we found that REVEL correctly predicts a high proportion of both LoF and GoF variants as pathogenic based on the REVEL score thresholds recommended by the tool authors. However, GoF variants are less likely to receive the highest REVEL scores, which would preclude the score from being used as strong evidence of pathogenicity in some cases.

## Data Availability

The list of variants used in this study are included in Supplementary Table 1.

## Supplementary Materials

Supplementary Materials Supplementary Table 1 contains the list of variants used in this study.
