# Supplementary material for: REVEL Is Better at Predicting Pathogenicity of Loss-of-Function than Gain-of-Function Variants
Source: Hum Mutat. 2023 Dec 4;2023:8857940. doi: 10.1155/2023/8857940 (PMC11918948; doi:10.1155/2023/8857940)
Supplement: Supplementary Materials — Supplementary Table 1 contains the list of variants used in this study. [file 8857940.f1.pdf]

Supplementary Table 1: Variants used to evaluate the performance of REVEL on loss of function (LoF) and gain of function (GoF) variants. Diabetes includes neonatal diabetes and maturity onset diabetes of the young (MODY). HI; hyperinsulinism. Nomenclature is given according to the following transcripts: *ABCC8* NM_001287174.1, *GCK* NM_000162.3 and *KCNJ11* NM_000525.4.

Gene	Protein change	Nucleotide change	Genomic location (GRCh37)	REVEL score	GoF or LoF	Phenotype
ABCC8	p.(Val86Ala)	c.257T>C	11:17496466A>G	0.794	GoF	Diabetes
ABCC8	p.(Val86Gly)	c.257T>G	11:17496466A>C	0.97	GoF	Diabetes
ABCC8	p.(Phe132Leu)	c.394T>C	11:17491666A>G	0.89	GoF	Diabetes
ABCC8	p.(Phe132Val)	c.394T>G	11:17491666A>C	0.808	GoF	Diabetes
ABCC8	p.(Glu208Lys)	c.622G>A	11:17483330C>T	0.669	GoF	Diabetes
ABCC8	p.(Asp209Glu)	c.627C>A	11:17483325G>T	0.739	GoF	Diabetes
ABCC8	p.(Gln211Lys)	c.631C>A	11:17483321G>T	0.617	GoF	Diabetes
ABCC8	p.(Leu213Arg)	c.638T>G	11:17483314A>C	0.72	GoF	Diabetes
ABCC8	p.(Leu225Pro)	c.674T>C	11:17483278A>G	0.769	GoF	Diabetes
ABCC8	p.(Thr229Ile)	c.686C>T	11:17483266G>A	0.894	GoF	Diabetes
ABCC8	p.(Val324Met)	c.970G>A	11:17482076C>T	0.895	GoF	Diabetes
ABCC8	p.(Leu451Pro)	c.1352T>C	11:17464840A>G	0.936	GoF	Diabetes
ABCC8	p.(Leu582Val)	c.1744C>G	11:17452434G>C	0.839	GoF	Diabetes
ABCC8	p.(Arg826Trp)	c.2476C>T	11:17434943G>A	0.855	GoF	Diabetes
ABCC8	p.(His1024Tyr)	c.3070C>T	11:17428530G>A	0.687	GoF	Diabetes
ABCC8	p.(Asn1123Asp)	c.3367A>G	11:17427076T>C	0.896	GoF	Diabetes
ABCC8	p.(Arg1183Trp)	c.3547C>T	11:17426072G>A	0.885	GoF	Diabetes
ABCC8	p.(Arg1183Gln)	c.3548G>A	11:17426071C>T	0.767	GoF	Diabetes
ABCC8	p.(Arg1314His)	c.3941G>A	11:17418790C>T	0.933	GoF	Diabetes
ABCC8	p.(Arg1380Cys)	c.4138C>T	11:17417462G>A	0.953	GoF	Diabetes
ABCC8	p.(Arg1380His)	c.4139G>A	11:17417461C>T	0.964	GoF	Diabetes
ABCC8	p.(Arg1380Leu)	c.4139G>T	11:17417461C>A	0.959	GoF	Diabetes
ABCC8	p.(Ile1425Val)	c.4273A>G	11:17417194T>C	0.772	GoF	Diabetes

ABCC8	p.(Gly7Arg)	c.19G>C	11:17498305C>G	0.954	LoF	HI
ABCC8	p.(Val21Asp)	c.62T>A	11:17498262A>T	0.863	LoF	HI
ABCC8	p.(Asn24Lys)	c.72C>A	11:17498252G>T	0.858	LoF	HI
ABCC8	p.(Arg74Trp)	c.220C>T	11:17496503G>A	0.976	LoF	HI
ABCC8	p.(Gly111Trp)	c.331G>T	11:17491729C>A	0.636	LoF	HI
ABCC8	p.(His125Gln)	c.375C>G	11:17491685G>C	0.888	LoF	HI
ABCC8	p.(Val187Asp)	c.560T>A	11:17485004A>T	0.815	LoF	HI
ABCC8	p.(Gly228Asp)	c.683G>A	11:17483269C>T	0.614	LoF	HI
ABCC8	p.(Asp310Asn)	c.928G>A	11:17482118C>T	0.833	LoF	HI
ABCC8	p.(Arg370Gly)	c.1108A>G	11:17474734T>C	0.644	LoF	HI
ABCC8	p.(Arg370Ser)	c.1110G>C	11:17474732C>G	0.559	LoF	HI
ABCC8	p.(Arg495Gln)	c.1484G>A	11:17464413C>T	0.872	LoF	HI
ABCC8	p.(Arg1215Trp)	c.3643C>T	11:17424218G>A	0.964	LoF	HI
ABCC8	p.(Arg1353His)	c.4058G>A	11:17418527C>T	0.934	LoF	HI
ABCC8	p.(Lys1374Arg)	c.4121A>G	11:17418464T>C	0.868	LoF	HI
ABCC8	p.(Ser1386Pro)	c.4156T>C	11:17417444A>G	0.979	LoF	HI
ABCC8	p.(Gly1478Val)	c.4433G>T	11:17415928C>A	0.88	LoF	HI
ABCC8	p.(Gly1479Arg)	c.4435G>A	11:17415926C>T	0.939	LoF	HI
ABCC8	p.(Arg1494Trp)	c.4480C>T	11:17415881G>A	0.93	LoF	HI
ABCC8	p.(Arg1494Gln)	c.4481G>A	11:17415880C>T	0.979	LoF	HI
ABCC8	p.(Glu1507Lys)	c.4519G>A	11:17415842C>T	0.963	LoF	HI
ABCC8	p.(Ile1512Thr)	c.4535T>C	11:17415826A>G	0.927	LoF	HI
ABCC8	p.(Arg1539Gln)	c.4616G>A	11:17414671C>T	0.877	LoF	HI
ABCC8	p.(Leu1544Pro)	c.4631T>C	11:17414656A>G	0.915	LoF	HI
GCK	p.(Ser64Tyr)	c.191C>A	7:44192917G>T	0.984	GoF	HI
GCK	p.(Thr65Ile)	c.194C>T	7:44192914G>A	0.82	GoF	HI
GCK	p.(Gly68Val)	c.203G>T	7:44192905C>A	0.993	GoF	HI

GCK	p.(Trp99Arg)	c.295T>C	7:44191938A>G	0.753	GoF	HI
GCK	p.(Tyr214Cys)	c.641A>G	7:44189397T>C	0.706	GoF	HI
GCK	p.(Val452Leu)	c.1354G>C	7:44184779C>G	0.601	GoF	HI
GCK	p.(Val455Met)	c.1363G>A	7:44184770C>T	0.853	GoF	HI
GCK	p.(Ala456Val)	c.1367C>T	7:44184766G>A	0.883	GoF	HI
GCK	p.(Gly44Ser)	c.130G>A	7:44192978C>T	0.962	LoF	Diabetes
GCK	p.(Gly44Asp)	c.131G>A	7:44192977C>T	0.995	LoF	Diabetes
GCK	p.(Tyr108His)	c.322T>C	7:44191911A>G	0.949	LoF	Diabetes
GCK	p.(Cys129Tyr)	c.386G>A	7:44190652C>T	0.97	LoF	Diabetes
GCK	p.(Phe150Ser)	c.449T>C	7:44190589A>G	0.983	LoF	Diabetes
GCK	p.(Asp160Asn)	c.478G>A	7:44190560C>T	0.712	LoF	Diabetes
GCK	p.(Asn180Lys)	c.540T>G	7:44189607A>C	0.751	LoF	Diabetes
GCK	p.(Val182Met)	c.544G>A	7:44189603C>T	0.937	LoF	Diabetes
GCK	p.(Ala188Thr)	c.562G>A	7:44189585C>T	0.933	LoF	Diabetes
GCK	p.(Arg191Trp)	c.571C>T	7:44189576G>A	0.936	LoF	Diabetes
GCK	p.(Ala201Ser)	c.601G>T	7:44189437C>A	0.929	LoF	Diabetes
GCK	p.(Val203Ala)	c.608T>C	7:44189430A>G	0.946	LoF	Diabetes
GCK	p.(Gly223Ser)	c.667G>A	7:44189371C>T	0.943	LoF	Diabetes
GCK	p.(Gly261Arg)	c.781G>A	7:44187331C>T	0.914	LoF	Diabetes
GCK	p.(Glu265Lys)	c.793G>A	7:44187319C>T	0.728	LoF	Diabetes
GCK	p.(Asp278Glu)	c.834C>A	7:44187278G>T	0.896	LoF	Diabetes
GCK	p.(Ser340Ile)	c.1019G>T	7:44186062C>A	0.792	LoF	Diabetes
GCK	p.(Met381Thr)	c.1142T>C	7:44185207A>G	0.93	LoF	Diabetes
GCK	p.(Arg392Cys)	c.1174C>T	7:44185175G>A	0.911	LoF	Diabetes
GCK	p.(Arg397Leu)	c.1190G>T	7:44185159C>A	0.925	LoF	Diabetes
KCNJ11	p.(Ser3Cys)	c.8C>G	11:17409631G>C	0.658	GoF	Diabetes
KCNJ11	p.(His46Tyr)	c.136C>T	11:17409503G>A	0.845	GoF	Diabetes

KCNJ11	p.(Arg50Gln)	c.149G>A	11:17409490C>T	0.831	GoF	Diabetes
KCNJ11	p.(Arg50Pro)	c.149G>C	11:17409490C>G	0.887	GoF	Diabetes
KCNJ11	p.(Glu51Ala)	c.152A>C	11:17409487T>G	0.907	GoF	Diabetes
KCNJ11	p.(Gln52Arg)	c.155A>G	11:17409484T>C	0.811	GoF	Diabetes
KCNJ11	p.(Gly53Ser)	c.157G>A	11:17409482C>T	0.794	GoF	Diabetes
KCNJ11	p.(Gly53Arg)	c.157G>C	11:17409482C>G	0.817	GoF	Diabetes
KCNJ11	p.(Gly53Asp)	c.158G>A	11:17409481C>T	0.841	GoF	Diabetes
KCNJ11	p.(Val59Met)	c.175G>A	11:17409464C>T	0.766	GoF	Diabetes
KCNJ11	p.(Val59Gly)	c.176T>G	11:17409463A>C	0.939	GoF	Diabetes
KCNJ11	p.(Leu164Pro)	c.491T>C	11:17409148A>G	0.973	GoF	Diabetes
KCNJ11	p.(Cys166Tyr)	c.497G>A	11:17409142C>T	0.954	GoF	Diabetes
KCNJ11	p.(Cys166Phe)	c.497G>T	11:17409142C>A	0.944	GoF	Diabetes
KCNJ11	p.(Ile167Leu)	c.499A>C	11:17409140T>G	0.828	GoF	Diabetes
KCNJ11	p.(Lys170Thr)	c.509A>C	11:17409130T>G	0.99	GoF	Diabetes
KCNJ11	p.(Lys170Arg)	c.509A>G	11:17409130T>C	0.946	GoF	Diabetes
KCNJ11	p.(Lys170Asn)	c.510G>C	11:17409129C>G	0.886	GoF	Diabetes
KCNJ11	p.(Glu179Ala)	c.536A>C	11:17409103T>G	0.945	GoF	Diabetes
KCNJ11	p.(Ile182Val)	c.544A>G	11:17409095T>C	0.786	GoF	Diabetes
KCNJ11	p.(Arg201Ser)	c.601C>A	11:17409038G>T	0.969	GoF	Diabetes
KCNJ11	p.(Arg201Cys)	c.601C>T	11:17409038G>A	0.977	GoF	Diabetes
KCNJ11	p.(Arg201His)	c.602G>A	11:17409037C>T	0.982	GoF	Diabetes
KCNJ11	p.(Arg201Leu)	c.602G>T	11:17409037C>A	0.986	GoF	Diabetes
KCNJ11	p.(Glu227Lys)	c.679G>A	11:17408960C>T	0.972	GoF	Diabetes
KCNJ11	p.(Glu229Lys)	c.685G>A	11:17408954C>T	0.981	GoF	Diabetes
KCNJ11	p.(Leu233Phe)	c.697C>T	11:17408942G>A	0.752	GoF	Diabetes
KCNJ11	p.(Val252Met)	c.754G>A	11:17408885C>T	0.918	GoF	Diabetes
KCNJ11	p.(Val252Gly)	c.755T>G	11:17408884A>C	0.97	GoF	Diabetes

KCNJ11	p.(Thr293Asn)	c.878C>A	11:17408761G>T	0.832	GoF	Diabetes
KCNJ11	p.(Ile296Leu)	c.886A>C	11:17408753T>G	0.834	GoF	Diabetes
KCNJ11	p.(Glu322Lys)	c.964G>A	11:17408675C>T	0.911	GoF	Diabetes
KCNJ11	p.(Tyr330Ser)	c.989A>C	11:17408650T>G	0.981	GoF	Diabetes
KCNJ11	p.(Gly334Asp)	c.1001G>A	11:17408638C>T	0.787	GoF	Diabetes
KCNJ11	p.(Arg34His)	c.101G>A	11:17409538C>T	0.965	LoF	HI
KCNJ11	p.(Gly40Asp)	c.119G>A	11:17409520C>T	0.988	LoF	HI
KCNJ11	p.(Phe55Leu)	c.165C>A	11:17409474G>T	0.924	LoF	HI
KCNJ11	p.(Thr62Met)	c.185C>T	11:17409454G>A	0.952	LoF	HI
KCNJ11	p.(Lys67Asn)	c.201G>C	11:17409438C>G	0.815	LoF	HI
KCNJ11	p.(Trp91Arg)	c.271T>C	11:17409368A>G	0.953	LoF	HI
KCNJ11	p.(Ala101Asp)	c.302C>A	11:17409337G>T	0.467	LoF	HI
KCNJ11	p.(Ser116Pro)	c.346T>C	11:17409293A>G	0.93	LoF	HI
KCNJ11	p.(Gly134Ala)	c.401G>C	11:17409238C>G	0.976	LoF	HI
KCNJ11	p.(Arg136Leu)	c.407G>T	11:17409232C>A	0.984	LoF	HI
KCNJ11	p.(Gly156Arg)	c.466G>A	11:17409173C>T	0.99	LoF	HI
KCNJ11	p.(Asp204Glu)	c.612C>A	11:17409027G>T	0.948	LoF	HI
KCNJ11	p.(Ala213Thr)	c.637G>A	11:17409002C>T	0.954	LoF	HI
KCNJ11	p.(Pro254Leu)	c.761C>T	11:17408878G>A	0.971	LoF	HI
KCNJ11	p.(His259Arg)	c.776A>G	11:17408863T>C	0.993	LoF	HI
KCNJ11	p.(Pro266Leu)	c.797C>T	11:17408842G>A	0.979	LoF	HI
KCNJ11	p.(Glu282Lys)	c.844G>A	11:17408795C>T	0.961	LoF	HI
KCNJ11	p.(Thr294Met)	c.881C>T	11:17408758G>A	0.958	LoF	HI
KCNJ11	p.(Arg301Gly)	c.901C>G	11:17408738G>C	0.946	LoF	HI
KCNJ11	p.(Arg301Cys)	c.901C>T	11:17408738G>A	0.94	LoF	HI
KCNJ11	p.(Arg301His)	c.902G>A	11:17408737C>T	0.972	LoF	HI
KCNJ11	p.(Arg301Pro)	c.902G>C	11:17408737C>G	0.984	LoF	HI
